# Pragmatic Comparison of Piperacillin/Tazobactam versus Carbapenems in Treating Patients with Nosocomial Pneumonia Caused by Extended-Spectrum β-Lactamase-Producing *Klebsiella pneumoniae*

**DOI:** 10.3390/antibiotics11101384

**Published:** 2022-10-10

**Authors:** Lei Zha, Xiang Li, Zhichu Ren, Dayan Zhang, Yi Zou, Lingling Pan, Shirong Li, Shanghua Chen, Boris Tefsen

**Affiliations:** 1 Intensive Care Unit, Conch Hospital of Anhui Medical University, Wuhu 241000, China; 2 Department of Biological Sciences, Xi’an Jiaotong-Liverpool University, Suzhou 215123, China; 3 Institute of Infection and Global Health, University of Liverpool, Liverpool L69 7BE, UK; 4 Postgraduate School, Wannan Medical College, Wuhu 241000, China; 5 Cardiology Department, The First Affiliated Hospital of Wannan Medical College, Wuhu 241000, China; 6 Pulmonary and Critical Care Department, The Second People’s Hospital of Wuhu, Wuhu 241000, China; 7 Intensive Care Unit, The Second People’s Hospital of Wuhu, Wuhu 241000, China; 8 Department of Molecular Microbiology, Utrecht University, 3584 CH Utrecht, The Netherlands; 9 Natural Sciences, Ronin Institute, Montclair, NJ 07043, USA

**Keywords:** carbapenem, ESBL, *Klebsiella pneumoniae*, nosocomial infection, pneumonia, piperacillin/tazobactam

## Abstract

The effectiveness of piperacillin/tazobactam for managing nosocomial pneumonia caused by extended-spectrum β-lactamase (ESBL)-producing *Enterobacteriaceae* is unknown. To answer this question, we conducted a retrospective cohort study in two tertiary teaching hospitals of patients admitted between January 2018 and July 2021 with a diagnosis of nosocomial pneumonia caused by ESBL-producing *K. pneumoniae* receiving either piperacillin/tazobactam or carbapenems within 24 h from the onset of pneumonia for at least 72 h. Clinical outcomes, including 28-day mortality and 14-day clinical and microbiological cure, were analyzed. Of the 136 total patients, 64 received piperacillin/tazobactam and 72 received carbapenems. The overall 28-day mortality was 19.1% (26/136). In the inverse probability of treatment weighted cohort, piperacillin/tazobactam therapy was not associated with worse clinical outcomes, as the 28-day mortality (OR, 0.82, 95% CI, 0.23–2.87, *p* = 0.748), clinical cure (OR, 0.94, 95% CI, 0.38–2.35, *p* = 0.894), and microbiological cure (OR, 1.10, 95% CI, 0.53–2.30, *p* = 0.798) were comparable to those of carbapenems. Subgroup analyses also did not demonstrate any statistical differences. In conclusion, piperacillin/tazobactam could be an effective alternative to carbapenems for treating nosocomial pneumonia due to ESBL-producing *K. pneumoniae* when the MICs are ≤8 mg/L.

## 1. Introduction

Infections caused by third-generation cephalosporin-resistant *Enterobacteriaceae* mediated mainly by the expression of extended-spectrum β-lactamases (ESBL) have increased significantly, thereby posing great challenges to clinicians by restricting the choice of antimicrobial agents [1,2]. Data from a report by the European Antimicrobial Resistance Surveillance Network in 2019 indicated that 15.1% of *Escherichia coli* and 31.7% of *Klebsiella pneumoniae* were resistant to third-generation cephalosporins [3], while the rate was reported to be as high as 61.3% for *E. coli* and 60.3% for *K. pneumoniae* in a Chinese study from 2018 [4]. The clinical impact of ESBL-producing *Enterobacteriaceae* has been well studied, as these pathogens cause worse clinical outcomes when compared with their non-ESBL-producing counterparts [5]. A recent meta-analysis including 84 studies with 22,030 patients reported increased attributable mortality by a factor of 1.75 (95% CI, 1.45–2.11) in bloodstream infections caused by ESBL-producing *Enterobacteriaceae* [6].

Carbapenems are typically the first-choice antibiotics recommended by clinical guidelines to treat infections caused by ESBL-producing *Enterobacteriaceae* [7,8]. They withstand hydrolysis by ESBL enzymes well and therefore present good activity against these pathogens [9,10,11]. A recent study that collected 7168 clinical isolates of *Enterobacteriaceae* from patients in the USA and Europe between 2016 and 2018 demonstrated a high susceptibility rate to meropenem at 97.6% in isolates with the ESBL genotype [12]. Similarly good in vitro activity was also described in a Chinese study, with the susceptibility rate to imipenem of ESBL-producing *E. coli* being 99.7% and that of ESBL-producing *K. pneumoniae* being 98% [13]. However, the increased use of carbapenems has led to the emergence of carbapenem-resistant bacteria, which places an added burden on public health [14,15]. Therefore, it is urgently necessary to find an effective carbapenem-sparing therapy for infections caused by ESBL-producing *Enterobacteriaceae*.

Classic β-lactam/β-lactamase inhibitor combinations, such as amoxicillin/clavulanate, ampicillin/sulbactam, cefoperazone/sulbactam, and piperacillin/tazobactam, usually demonstrate good in vitro activity against ESBL-producing *Enterobacteriaceae* when these pathogens do not possess other antimicrobial resistant mechanism(s) [10,12]. Among the available classic β-lactam/β-lactamase inhibitor combinations, piperacillin/tazobactam is one of the most interesting carbapenem-sparing therapies against infections caused by ESBL-producing *Enterobacteriaceae* [9,16]. A recent antimicrobial resistance surveillance study indicated that the susceptibility of *Enterobacteriaceae* with the ESBL genotype to piperacillin/tazobactam was as high as 71.4%, compared to only 18% and 11.9% for amoxicillin/clavulanate and amoxicillin/sulbactam, respectively [12]. Clinical studies have demonstrated the comparable effectiveness of piperacillin/tazobactam and carbapenems in the treatment of urinary tract infections [17,18,19]. However, the efficacy of piperacillin/tazobactam in treating bacteremia caused by ESBL-producing *Enterobacteriaceae* is still uncertain, as some studies have indicated comparable effectiveness [20,21,22,23,24] while some have demonstrated inferiority [25,26,27].

Evidence supporting the use of β-lactam/β-lactamase inhibitor combinations in the treatment of bloodstream infections due to ESBL-producers is primarily based on the INCREMENT study, a multinational, preregistered cohort study with a large sample size, in which the 30-day mortality did not show any statistical differences between β-lactam/β-lactamase inhibitor combinations (amoxicillin/clavulanate and piperacillin/tazobactam) and carbapenems (ertapenem, meropenem, imipenem, and doripenem) in both empiric and definitive therapy cohorts [20]. Moreover, there were no differences detected between amoxicillin/clavulanate and piperacillin/tazobactam versus carbapenems in the subgroup analysis, indicating support for the use of the β-lactam/β-lactamase inhibitor combination to minimize carbapenem use [20]. Similar results were subsequently published in two meta-analyses [28,29].

By contrast, the MERINO trial, which compared piperacillin/tazobactam with meropenem in the treatment of bloodstream infections caused by ceftriaxone-resistant *E. coli* or *K. pneumoniae* did not support the use of piperacillin/tazobactam [27]. The 30-day mortality in patients receiving piperacillin/tazobactam was statistically higher than that of those receiving meropenem (12.3% vs. 3.7%) [27]. However, a post hoc analysis by the same group of authors, which involved re-performing the antimicrobial susceptibility testing with the referred broth microdilution methods, did not support the inferiority of piperacillin/tazobactam [21]. It found that there were a significant proportion of piperacillin/tazobactam non-susceptible strains included in the MERINO trial, and when those non-susceptible strains were excluded the between group difference in 30-day mortality was reduced to 5% (95% CI, −1% to 10%) [21]. Therefore, it can be concluded that piperacillin/tazobactam is as effective as meropenem in treating bacteremia caused by piperacillin/tazobactam-susceptible ESBL-producers.

Because there have not been any clinical studies dedicated to the therapeutic options for pneumonia caused by ESBL-producing *Enterobacteriaceae*, guidelines [7,8] against using piperacillin/tazobactam in such cases has been largely based on the original findings by Harris et al. [27] in the MERINO trial. However, when considering the aforementioned factors of the MERINO trial described two years later by Henderson et al. [21], it is reasonable to hypothesize that piperacillin/tazobactam might be an effective alternative to carbapenems for treating pneumonia caused by ESBL-producers if susceptibility is established. Therefore, we conducted this retrospective cohort study to test this hypothesis by treating patients with nosocomial pneumonia caused by ESBL-producing *K. pneumoniae* with either carbapenems or piperacillin/tazobactam.

## 2. Methods

### 2.1. Study Design

This is a retrospective, observational cohort study conducted in two tertiary teaching hospitals in Wuhu, Anhui, China (the First Affiliated Hospital of Wannan Medical College and the Second People’s Hospital of Wuhu), which in total have around 5000 beds for inpatients. The medical records of patients diagnosed with nosocomial pneumonia caused by ESBL-producing *K. pneumoniae* were reviewed from January 2018 to July 2021. The study was approved by the ethics committee of Xi’an Jiaotong-Liverpool University (reference number 19-01-05) and the institutional review board in each participating hospital, and informed consent was waived due to the retrospective nature of the study.

### 2.2. ESBL-Producing K. pneumoniae Nosocomial Pneumonia

Nosocomial pneumonia (including hospital-acquired pneumonia (HAP) and ventilator-associated pneumonia (VAP)) was diagnosed according to the 2016 clinical practice guideline by the Infectious Diseases Society of America and the American Thoracic Society [8]. The diagnosis of pneumonia was made based on a newly developed or progressive lung infiltration or consolidation on chest radiographs plus two or more of the following criteria: new onset or worsening cough; temperature >38 °C or <35 °C; leukocyte count > 10 × 10^12^/L or <4 × 10^12^/L; purulent sputum or endotracheal aspirate; hypoxemia or worsening oxygenation that required increment of ventilation support. VAP was defined as pneumonia occurring in patients receiving invasive mechanical ventilation ≥48 h, and HAP was defined as pneumonia occurring ≥48 h after hospitalization, excluding patients with VAP. Pathogens responsible for the episode of nosocomial pneumonia were determined by semiquantitative culture of qualified respiratory specimens [30]. Microorganism identification and ESBL-phenotype determination were performed with a Vitek 2 system (bioMérieux, Lyon, France). Susceptibility to piperacillin/tazobactam was measured with the microdilution method and determined according to the breakpoint recommended by EUCAST (MIC ≤ 8 mg/L) [31]. In brief, the Vitek 2 ESBL test is a rapid detection tool with good specificity (99.7%) and sensitivity (98.1%), which is based on the simultaneous assessment of the inhibitory effects of cefepime, cefotaxime, and ceftazidime, alone and in the presence of clavulanic acid, similar to what is recommended by the Clinical and Laboratory Standards Institute (CLSI) [32,33].

### 2.3. Participants

Patients aged >18 years with a diagnosis of nosocomial pneumonia caused by ESBL-producing *K. pneumoniae* receiving either piperacillin/tazobactam or carbapenems (either imipenem or meropenem in this study) within 24 h from the onset of pneumonia and for at least the subsequent 72 h were eligible. Piperacillin/tazobactam was administrated as 4.5 g every 6 h by extended infusion. Imipenem or meropenem was administrated as 1 g every 8 h intravenously without an extended infusion. Dosage adjustments were made based on renal function. Patients meeting the following criteria were excluded: received both carbapenems and piperacillin/tazobactam during the pneumonia course; combined with other antibiotics; pneumonia was polymicrobial; ESBL-producing *K. pneumonia* was non-susceptible to piperacillin/tazobactam (defined as MIC > 8 mg/L according to EUCAST breakpoint [31]) or carbapenems (defined as MIC of imipenem > 1 mg/L); patients with concurrent infections other than pneumonia that required other antimicrobials in addition to piperacillin/tazobactam or carbapenems, such as intra-abdominal infections, etc. Only the first episode was included in this study in cases where patients experienced more than one episode of nosocomial pneumonia caused by ESBL-producing *K. pneumoniae*.

### 2.4. Outcomes and Definitions

The primary endpoint was 28-day all-cause mortality after the onset of nosocomial pneumonia. Secondary outcomes were clinical cure and microbiological cure. Clinical cure was defined as complete resolution of all signs and symptoms of pneumonia or such improvement of patients that antibiotics were stopped at day 14 after the onset of pneumonia. Microbiological cure was defined as the absence of ESBL-producing *K. pneumoniae* in the culture of specimens collected within two days before or after the visit time point on day 14 after the onset of pneumonia. Patients who died or were discharged within 14 days were excluded from the microbiological cure analysis.

### 2.5. Data Extraction

Data were collected from medical records and included patients’ demographics (age, gender), reasons for hospitalization, preexisting medical conditions, severity of disease at the time of nosocomial pneumonia onset (Acute Physiology and Chronic Health Evaluation II (APACHE II) score [34]), type of pneumonia (HAP, VAP), duration of antibiotic therapy for pneumonia, clinical and microbiological outcomes, and the 28-day mortality. For patients discharged from the hospital earlier than 28 days after the onset of nosocomial pneumonia, information on 28-day mortality was obtained from the one-month follow-up records.

### 2.6. Statistical Analysis

Continuous variables were summarized as medians and interquartile ranges. Categorical variables were described as count and percentage. The differences between patients receiving piperacillin/tazobactam or carbapenems were analyzed with the Chi-square test or Fisher’s exact test for categorical variables and Wilcoxon’s rank-sum test for continuous variables.

To balance the baseline differences in the two groups, an inverse probability of treatment weighting was performed. The propensity score was estimated using a non-parsimonious multivariable logistic regression model with receiving piperacillin/tazobactam as the dependent variable and the baseline characteristics in the two groups with a standardized mean difference of more than 0.2 and those preexisting medical conditions (decided a priori) as covariates. The final variables were gender, shock, APACHE II score, immunocompromised status, chronic kidney disease, chronic liver disease, chronic heart disease, chronic respiratory disease, malignancy, cerebrovascular disease, and diabetes mellitus. Weights were stabilized to reduce the influence of extreme weights if needed. The characteristics in the inverse probability of treatment weighted cohort were considered balanced if the standardized mean difference values were less than 0.1. Odds ratios (OR) and 95% confidence intervals (CI) for the 28-day mortality and clinical cure were then estimated using the weighted cohort that was adjusted for age, type of pneumonia, and APACHE II score. The OR and 95% CI for the microbiological cure were calculated in the microbiologically evaluable population by multivariable regression while adjusting for the same covariates.

Subgroup analyses of the primary outcome were also performed by stratifying patients by age (>65 years, or ≤65 years), APACHE II score (>15, or ≤15), and type of pneumonia (HAP, or VAP). The OR and 95% CI in each subgroup were estimated by adjusting for age and APACHE II score in the multivariable regression analysis. Two-tailed *p* < 0.05 was considered statistically significant. All the statistical analyses were performed with R software version 3.6.2 (R Foundation for Statistical Computing).

## 3. Results

### 3.1. Patient Characteristics

**Figure 1:**
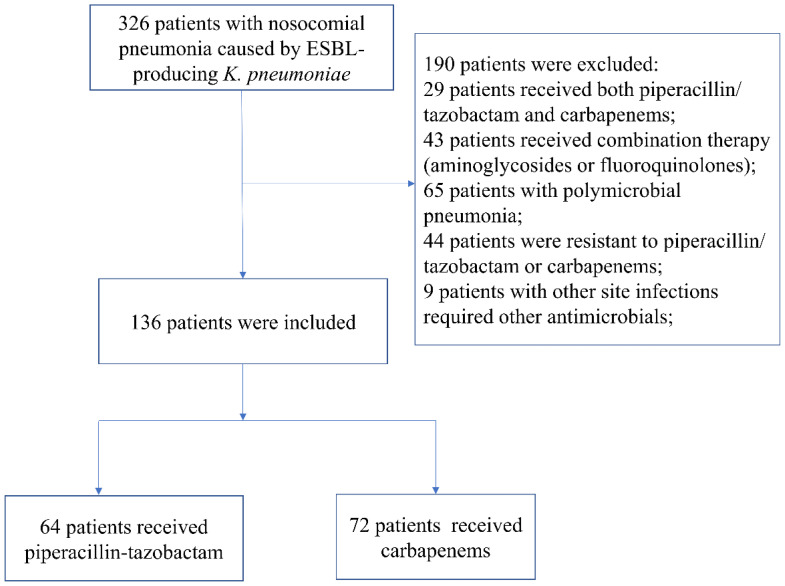
Flowchart of the study inclusion process.

A total of 326 patients were diagnosed with nosocomial pneumonia caused by ESBL-producing *K. pneumoniae* meeting the inclusion criteria during the study period, 190 of whom met the exclusion criteria. The remaining 136 patients were included in the final analysis, with 64 patients being treated with piperacillin/tazobactam and 72 being treated with carbapenems (58 patients with meropenem and 14 patients with imipenem) (Figure 1). All strains isolated in the present study had a MIC of piperacillin/tazobactam ≤ 8 mg/L; 57.4% (78/136) of *K. pneumoniae* strains had a piperacillin/tazobactam MIC of 4 mg/L, and 42.6% (58/136) had a piperacillin/tazobactam MIC of 8 mg/L. Patients with an MIC ≤ 4 mg/L were more likely to receive piperacillin/tazobactam rather than carbapenems (76.6% vs. 40.3%, *p* < 0.01).

The median age of the included patients was 68 years (IQR 55–76), and 108 (79.4%) were male. Most patients had at least one comorbidity, with cerebrovascular disease (57, 41.9%) and hypertension (54, 39.7%) being the most reported. Moreover, 36 (26.5%) patients had a history of malignancy, and 14 (10.3%) patients were immunocompromised. The median APACHE II score was 14 (IQR 11–19), and there was no statistical significance between patients receiving piperacillin/tazobactam and carbapenems. The reasons for hospitalization included both internal and surgical diseases, among which stroke, respiratory failure, and scheduled surgery were the most documented. VAP was the diagnosis for 43 (31.6%) patients in the cohort, and 93 (68.4%) patients were diagnosed with HAP. The average duration of the target antibiotic therapy in the whole cohort was 8 days (IQR 5–12.25) and did not differ between patients receiving piperacillin/tazobactam and carbapenems. Other baseline characteristics were similar between the two groups (Table 1).

### 3.2. Inverse Probability of Treatment Weighted Cohort

**Figure 2:**
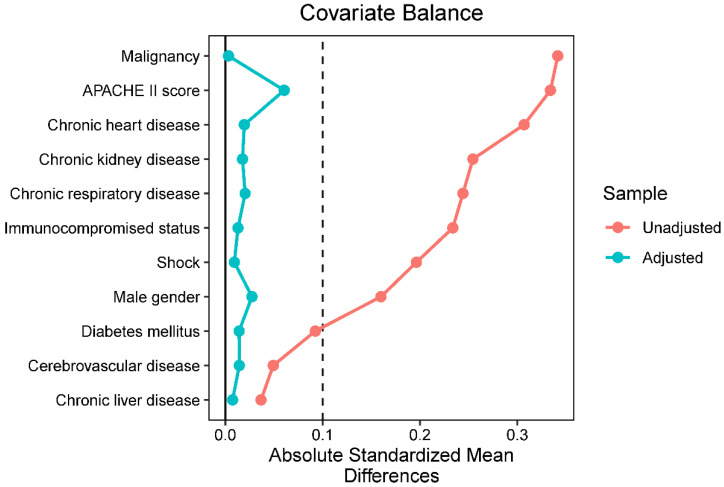
Standardized mean differences (SMD) between patients receiving piperacillin/tazobactam and carbapenems. The red dots represent the SMD in the whole unweighted cohort; the blue dots represent the SMD in the weighted cohort after the inverse probability of treatment weighting.

In China, carbapenems and piperacillin/tazobactam are both recommended in the clinical guidelines to treat pneumonia caused by ESBL-producing *Enterobacteriaceae* [35], and the attending doctors are responsible for the choice of which antibiotic to use, which might lead to indication bias in the present study. Although the baseline characteristics between the two groups were similar in the original cohort, we still conducted an inverse probability of treatment weighting in this study, which is one of the most popular methods to control confounding by indication in retrospective studies [36,37]. After weighting, the absolute standardized mean differences of variables of interest were lower than 0.1, indicating a similar distribution of observed covariates in the two groups (Figure 2).

### 3.3. Outcomes

**Figure 3:**
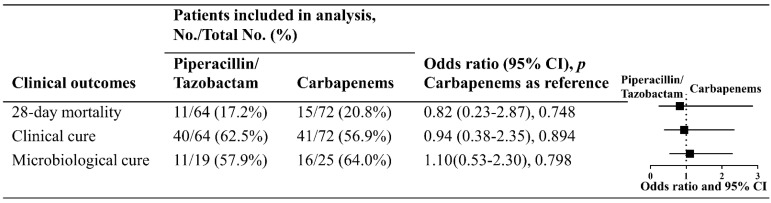
Clinical outcomes in patients with nosocomial pneumonia caused by ESBL-producing *K. pneumonia*. In the weighted cohort, the 28-day mortality was 17.4% vs. 18.5%, and the clinical cure was 63.8% vs. 62.2% in patients receiving piperacillin/tazobactam and carbapenems, respectively. The odds ratio and 95% confidence intervals for the 28-day mortality and clinical cure were calculated in the weighted cohort adjusting for age, APACHE II score, and type of pneumonia. The OR and 95% CI for the microbiological cure were estimated in the microbiologically evaluable cohort.

There were 26 (19.1%) patients who died within 28 days from the onset of pneumonia in the whole population; 11 (17.2%) patients in the piperacillin/tazobactam group and 15 (20.8%) patients in the carbapenem group. Receiving piperacillin/tazobactam was not associated with higher 28-day mortality than receiving carbapenems (OR, 0.82, 95% CI, 0.23–2.87, *p* = 0.748) according to the inverse probability of treatment weighted cohort adjusted for age, APACHE II score, and type of pneumonia. The overall clinical cure rate was 59.6% (81/136) at day 14 without any statistical differences between the two groups (62.5% (40/64) vs. 56.9% (41/72), respectively; OR, 0.94, 95% CI, 0.38–2.35, *p* = 0.894). To determine microbiological cure, 44 patients were included in the final analysis; of these, 57.9% (11/19) in the piperacillin/tazobactam group and 64% (16/25) in the carbapenem group were microbiologically cured on the follow-up visit (OR, 1.10, 95% CI, 0.53–2.30, *p* = 0.798) (Figure 3).

**Figure 4:**
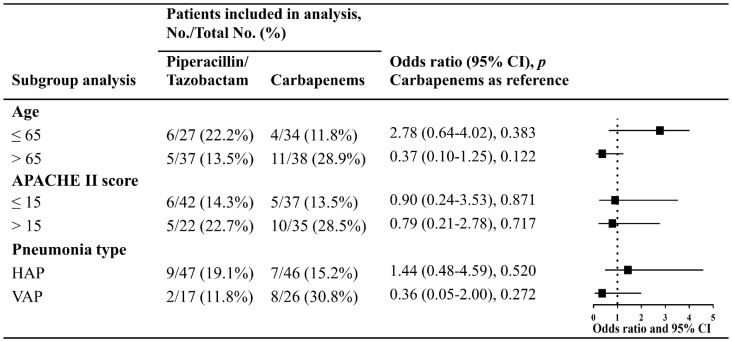
Subgroup analysis of the primary outcome in patients with nosocomial pneumonia caused by ESBL-producing *K. pneumonia*. The odds ratio and 95% confidence intervals for the 28-day mortality in each subgroup were estimated using carbapenems as the reference and adjusted for age and APACHE II score. HAP, hospital-acquired pneumonia, not including patients with VAP; VAP, ventilator-associated pneumonia; APACHE II score, Acute Physiology and Chronic Health Evaluation II score.

Moreover, subgroup analyses that involved stratifying patients by age (>65 years or ≤65 years), APACHE II score (>15 or ≤15), and type of pneumonia (HAP or VAP) did not indicate any statistical differences in the 28-day mortality between patients receiving piperacillin/tazobactam and those receiving carbapenems (Figure 4).

## 4. Discussion

Although many studies have assessed the effectiveness of piperacillin/tazobactam at treating various infections caused by ESBL-producing *Enterobacteriaceae*, there have not been any published studies specifically evaluating its efficacy at treating pneumonia caused by these pathogens. In the present study, we assessed the effectiveness of piperacillin/tazobactam at treating patients with nosocomial pneumonia due to ESBL-producing *K. pneumoniae* in comparison with carbapenems. The results suggest that piperacillin/tazobactam might be an effective alternative to carbapenems in treating such infections, as it resulted in similar 28-day mortality and 14-day clinical and microbiological cure. Clearly, our results differ from some studies that came to negative conclusions regarding the effectiveness of piperacillin/tazobactam in the treatment of bacteremia due to ESBL-producing *Enterobacteriaceae* (or ceftriaxone-resistant bacteria), which has been summarized and discussed in reviews [38,39,40,41,42]. Next, two important factors that might contribute to the different outcomes of such studies, i.e., the MIC breakpoints of piperacillin/tazobactam and the dosing/administration model used, will be discussed.

Which breakpoint of MIC for piperacillin/tazobactam is used to interpret the results of antimicrobial susceptibility testing against ESBL-producers matters greatly. The susceptible breakpoint of piperacillin/tazobactam in EUCAST is ≤8 mg/L, while it is ≤16 mg/L in CLSI [31,33]. Accordingly, ESBL-producers with an MIC between 8 and 16 mg/L have been included in studies using the CLSI breakpoint while they have been excluded in studies using the EUCAST breakpoint. The inclusion of patients with technical uncertainty (8 to 16 mg/L) who were subsequently treated with piperacillin/tazobactam would have probably affected clinical outcomes. A pharmacokinetic and pharmacodynamic (PK/PD) study indicated that the success rate of piperacillin/tazobactam (4 g every 6 h) achieving the target against ESBL-producers was 99% when the MIC of ESBL-producers was ≤8 mg/L, while the success rate decreased to 57% when the MIC reached 16 mg/L [43]. Clinical studies also suggest that the MIC to piperacillin/tazobactam in ESBL-producers was negatively associated with clinical outcomes. In a retrospective study that included patients with bacteremia due to ESBL-producing *E. coli*, the mortality rate was 4.5% in patients infected with strains that had an MIC to piperacillin/tazobactam ≤4.5 mg/L, while mortality was significantly increased to 23% in those infected by strains with an MIC ≥ 8 mg/L [44]. The post hoc analysis of the MERINO trial demonstrated a similar trend [21]. In patients with bacteremia caused by ceftriaxone-resistant *E. coli* or *K. pneumoniae* that did not originate from urinary tract infections, the mortality rate in patients receiving piperacillin/tazobactam was 27.3% when the MIC was ≤8 mg/L but increased to 71.4% when the MIC exceeded 8 mg/L [21]. Moreover, a retrospective study including patients with bacteremia caused by cefotaxime non-susceptible *E. coli* and *K. pneumonia* with a MIC to piperacillin/tazobactam ≤ 8 mg/L (70.7% patients infected by strains with an MIC of ≤4 mg/L, 29.3% patients by strains with an MIC of ≤8 mg/L) indicated comparable outcomes between piperacillin/tazobactam and carbapenems [45]. By contrast, another study comprising patients with bacteremia due to ESBL-producing bacteria with a higher MIC distribution (all ≤16 mg/L, 39% ≤4 mg/L, 46% ≤8 mg/L, and 14% ≤16 mg/L) demonstrated a worse clinical outcome in patients empirically receiving piperacillin/tazobactam [25]. Together with the results found in our study, where the EUCAST cut-off was used, it is reasonable to recommend restricting piperacillin/tazobactam use to ESBL-producers with an MIC of ≤8 mg/L.

A second factor that affects clinical outcomes is the dosing and administration model of piperacillin/tazobactam. Since piperacillin/tazobactam is a time-dependent antibiotic combination, the antimicrobial activity depends on the percentage of the dosing interval that the free drug concentration is maintained above the MIC of the target pathogen (*f*TMIC) [46]. PK/PD studies indicated that, compared with intermittent administration of 4 g of piperacillin/tazobactam every 8 *h*, those using 4.5 g of piperacillin/tazobactam every 6 h or by continuous infusion had higher success rates for achieving the probability of attainment for 50% and 100% *f*TMIC [47,48]. A systematic review and meta-analysis comparing the prolonged infusion of piperacillin/tazobactam with intermittent infusion in severely ill patients indicated that the prolonged infusion was associated with 1.46-times lower odds of mortality (95% CI, 1.20–1.77) [49]. Moreover, a retrospective study including patients with bacteremia due to ESBL-producers receiving different doses of piperacillin/tazobactam illustrated the opposite result [25]. In the subgroup analysis, patients receiving 4.5 g of piperacillin/tazobactam every 6 h did not show any difference in mortality compared with those receiving carbapenems; in contrast, the adjusted death rate was 1.92 times higher for patients receiving piperacillin/tazobactam when compared with those using carbapenems in the whole population, as 61% of patients were receiving 3.375 g of piperacillin/tazobactam every 6 h [25]. Therefore, the dose and administration model of piperacillin/tazobactam is essential to maintain favorable outcomes. Although the post hoc analysis of the MERINO study supports that intermittent infusion of piperacillin/tazobactam every 6 h was as effective as carbapenems when the pathogens were truly susceptible to it [21], considering the inoculum effect in the lung [50], we still recommend using piperacillin/tazobactam 4.5 g every 6 h by extended infusion (3 to 4 h) or continuous infusion for pneumonia, as was carried out in our study.

Despite the promising results of piperacillin/tazobactam demonstrated in this study, using this combination to treat ESBL-producing infections still needs to be assessed with great caution. The inaccuracy in piperacillin/tazobactam susceptibility determined by automatic systems in clinical practice is the primary concern [51], as was demonstrated by the post hoc analysis of the MERINO trial [21]. A considerable proportion of isolates were in fact not susceptible to piperacillin/tazobactam by broth microdilution but were categorized as piperacillin/tazobactam susceptible using automatic methods, which subsequently led to the failure of piperacillin/tazobactam therapy [21,27]. The inaccurate susceptibility of piperacillin/tazobactam in these ESBL-producing pathogens was due to the coharboring OXA-1 (oxacillinase-1) [51]. Studies have illustrated that pathogens coharboring OXA-1 and ESBL may show false susceptibility to piperacillin/tazobactam when measured with the Vitek 2 automatic system [52] or strip-gradient test (Etest) [21]. Nevertheless, it is still possible to account for this disadvantage in clinical practice. Isolates coharboring OXA-1 and ESBL that were associated with elevated piperacillin/tazobactam MICs [21] typically had the MIC of piperacillin/tazobactam at 8 to 16 mg/L [52], the area of technical uncertainty, a concept introduced by the EUCAST to account for the challenge of test variability [31]. Therefore, restricting the use of piperacillin/tazobactam to isolates with an MIC < 8 mg/L, the lower breakpoint of piperacillin/tazobactam suggested by EUCAST, makes it less likely to include clinically relevant OXA-1 strains in piperacillin/tazobactam therapy.

Apart from the coharboring OXA-1, the coexistence of AmpCs (Ambler Class C β-lactamases) in ESBL-producing bacteria is another concern when using piperacillin/tazobactam. Studies have reported that a significant proportion of *Enterobacteriaceae* coharbor both AmpC and ESBL [21,53]. AmpCs typically cause resistance against tazobactam, thereby also diminishing the efficacy of piperacillin in such pathogens [54]. As AmpCs are not routinely tested in clinical practice, this might add uncertainty to clinical decisions to use piperacillin/tazobactam in ESBL-producing bacterial infections. However, from the perspective of clinicians, it is very unlikely that they would use piperacillin/tazobactam in isolates coharboring both ESBL and clinically relevant AmpCs when the choice of antibiotics was made based on the results of antimicrobial susceptibility testing. Enterobacterales with plasmid-mediated *ampC* or derepressed chromosomal *ampC* usually express AmpC at a clinically relevant level and cause resistance against piperacillin/tazobactam [54,55]; in contrast, strains harboring chromosomal *ampC* but still exhibiting susceptibility to piperacillin/tazobactam generally express the AmpC at a very low level and they usually do not cause a clinical issue, similar to the chromosomal *ampC* in many *E. coli* strains [54,56]. Moreover, both piperacillin and tazobactam are weak inducers of AmpC enzymes [57]. Clinical studies have already demonstrated that piperacillin/tazobactam resulted in similar clinical outcomes to carbapenems when treating infections caused by ESBL-producing pathogens that expressed AmpC but are still susceptible to piperacillin/tazobactam [58,59]. Taken together, when considering using piperacillin/tazobactam to treat ESBL-producing infections, the accurate phenotypical susceptibility needs to be determined on top of the genomic background of the clinical isolates.

There are several limitations in the present study. First, it has a small sample size and is a retrospective study. Bias and confounders might still affect the final analysis despite implementing the propensity score weighting. Second, ESBL-production was determined phenotypically by the automatic Vitek 2 system instead of by the referred methods [33]. Although studies have indicated the excellent sensitivity and specificity of the Vitek 2 system [32,60], it still might include false-positive isolates in this study, hence skewing the results in favor of piperacillin/tazobactam. Third, the distribution of ESBL enzymes varies geographically [10,61]; thus, without knowing the genomic background, the results in this study might not be generalizable. Fourth, all patients included in this study displayed mild to moderate pneumonia, and only 11% (15/136) of patients had an APACHE II score >15. Thus, it is unclear how well piperacillin/tazobactam would function in severe patients. Fifth, with a meager rate of blood culture implemented in the study cohort, we did not incorporate the impact of bacteremia in the final analysis. Having this analysis would have strengthened the interpretation of clinical results, as it is known that bacteremia is an independent risk factor for mortality in patients with pneumonia [62]. Sixth, we only included patients receiving piperacillin/tazobactam or carbapenems starting from the onset of pneumonia; those using piperacillin/tazobactam only for definitive therapy were excluded. Therefore, we were unable to draw any conclusions regarding the efficacy of piperacillin/tazobactam definitive therapy in nosocomial pneumonia due to ESBL-producers. Seventh, in the present study, the economic cost of the two regimens was not incorporated in the final analysis; therefore, which regimen possesses a financial advantage is still unknown. Despite these limitations, to the best of our knowledge, this is the only study to date specifically focused on assessing the clinical effectiveness of piperacillin/tazobactam in the treatment of nosocomial pneumonia due to ESBL-producing *K. pneumonia*, and thus this work contributes towards using the most appropriate carbapenem-sparing therapy.

## 5. Conclusions

Piperacillin/tazobactam might be an effective alternative to carbapenems in treating nosocomial pneumonia caused by ESBL-producing *K. pneumoniae*. It was not associated with worse clinical outcomes compared with carbapenems. When considering using piperacillin/tazobactam to treat pneumonia caused by ESBL-producing *K. pneumoniae*, we recommend restricting its use to the extended or continuous infusion of 4.5 g every 6 h to treat patients infected by strains with an MIC of ≤8 mg/L, especially for those determined by automatic methods. In light of the limitations in the present study, appropriately powered and well-designed randomized controlled trials are required to confirm these findings.

## Figures and Tables

**Table 1:** Characteristics of patients with nosocomial pneumonia caused by ESBL-producing *K. pneumoniae*.

Variable	Original Cohort			Inverse Probability of Treatment Weighted Cohort			
	Piperacillin/tazobactam, *n* = 64, 47%	Carbapenems, *n* = 72, 53%	*p*	Piperacillin/Tazobactam, 50.5%	Carbapenems, 49.5%	*p*	Standardized Mean Differences
Age, years, median (IQR)	68 (56–75)	68 (54–76)	0.929	69 (56–75)	68 (53–77)	0.911	0.006
Male Gender, *n* (%)	53 (82.8)	55 (76.4)	0.476	77.6	78.7	0.884	0.028
Reasons for Admission, *n* (%)			0.051			0.181	
Traumatic Brain Injury	0 (0.0)	10 (13.9)		0.0	12.0		
Coronary Heart Disease	2 (3.1)	7 (9.7)		6.1	7.8		
Cancer Therapy	9 (14.1)	5 (6.9)		10.2	10.6		
Respiratory Failure	17 (26.5)	10 (13.9)		26.3	12.3		
Renal Failure	2 (3.1)	3 (4.2)		3.2	3.8		
Sepsis	1 (1.6)	2 (2.8)		1.7	2.0		
Stroke	15 (23.4)	20 (27.8)		22.9	29.8		
Scheduled Surgery	14 (21.9)	12 (16.7)		22.5	18.1		
Trauma	4 (6.2)	3 (4.2)		7.2	3.6		
Preexisting Medical Conditions, *n* (%)							
Immunocompromised Status	9 (14.1)	5 (6.9)	0.280	10.2	10.6	0.942	0.013
Hypertension	24 (37.5)	30 (41.7)	0.749	37.6	41.9	0.631	0.088
Cerebrovascular Disease	26 (40.6)	31 (43.1)	0.910	43.1	42.4	0.936	0.015
Diabetes Mellitus	12 (18.8)	11 (15.3)	0.757	17.1	16.6	0.938	0.014
Malignancy	22 (34.4)	14 (19.4)	0.076	25.4	25.5	0.985	0.003
Chronic Respiratory Disease	8 (12.5)	4 (5.6)	0.262	8.8	8.0	0.879	0.028
Chronic Kidney Disease	6 (9.4)	13 (18.1)	0.226	14.3	14.3	0.989	0.003
Chronic Liver Disease	6 (9.4)	6 (8.3)	1.000	8.5	6.4	0.617	0.081
Chronic Heart Disease	7 (10.9)	16 (22.2)	0.128	17.6	17.1	0.948	0.013
Shock	5 (7.8)	10 (13.9)	0.393	10.2	10.6	0.938	0.015
APACHE II score, median (IQR)	13 (10–17)	15 (11–20)	0.071	14 (11–19)	14 (10–19)	0.772	0.044
Nosocomial Pneumonia, *n* (%)			0.312			0.793	0.049
With Mechanical Ventilation	17 (26.6)	26 (36.1)		34.2	31.9		
Without Mechanical Ventilation	47 (73.4)	46 (63.9)		65.8	68.1		
Antibiotic duration, day, median (IQR)	9 (6–13)	7 (5–10.25)	0.160	9 (6–13)	7 (5–10.25)	0.073	0.274

## Data Availability

The datasets generated and analyzed during the current study are available from the corresponding author (Lei Zha) upon reasonable request.
